# Theoretical framework and experimental solution for the air−water interface adsorption problem in cryoEM

**DOI:** 10.52601/bpr.2023.230008

**Published:** 2023-08-31

**Authors:** Joon S. Kang, Xueting Zhou, Yun-Tao Liu, Kaituo Wang, Z. Hong Zhou

**Affiliations:** 1 Department of Microbiology, Immunology & Molecular Genetics, University of California, Los Angeles (UCLA), Los Angeles, CA 90095, USA; 2 Molecular Biology Institute, UCLA, Los Angeles, CA 90095, USA; 3 California NanoSystems Institute, UCLA, Los Angeles, CA 90095, USA

**Keywords:** CryoEM, CryoET, Sample preparation, Air–water interface adsorption, Surfactant, Surface energy

## Abstract

As cryogenic electron microscopy (cryoEM) gains traction in the structural biology community as a method of choice for determining atomic structures of biological complexes, it has been increasingly recognized that many complexes that behave well under conventional negative-stain electron microscopy tend to have preferential orientation, aggregate or simply mysteriously “disappear” on cryoEM grids. However, the reasons for such misbehavior are not well understood, which limits systematic approaches to solving the problem. Here, we have developed a theoretical formulation that explains these observations. Our formulation predicts that all particles migrate to the air–water interface (AWI) to lower the total potential surface energy-rationalizing the use of surfactant, which is a direct solution to reduce the surface tension of the aqueous solution. By performing cryogenic electron tomography (cryoET) on the widely-tested sample, GroEL, we demonstrate that, in a standard buffer solution, nearly all particles migrate to the AWI. Gradually reducing the surface tension by introducing surfactants decreased the percentage of particles exposed to the surface. By conducting single-particle cryoEM, we confirm that suitable surfactants do not damage the biological complex, thus suggesting that they might provide a practical, simple, and general solution to the problem for high-resolution cryoEM. Applying this solution to a real-world AWI adsorption problem involving a more challenging membrane protein, namely, the ClC-1 channel, has resulted in its near-atomic structure determination using cryoEM.

## INTRODUCTION

**Figure 1:**
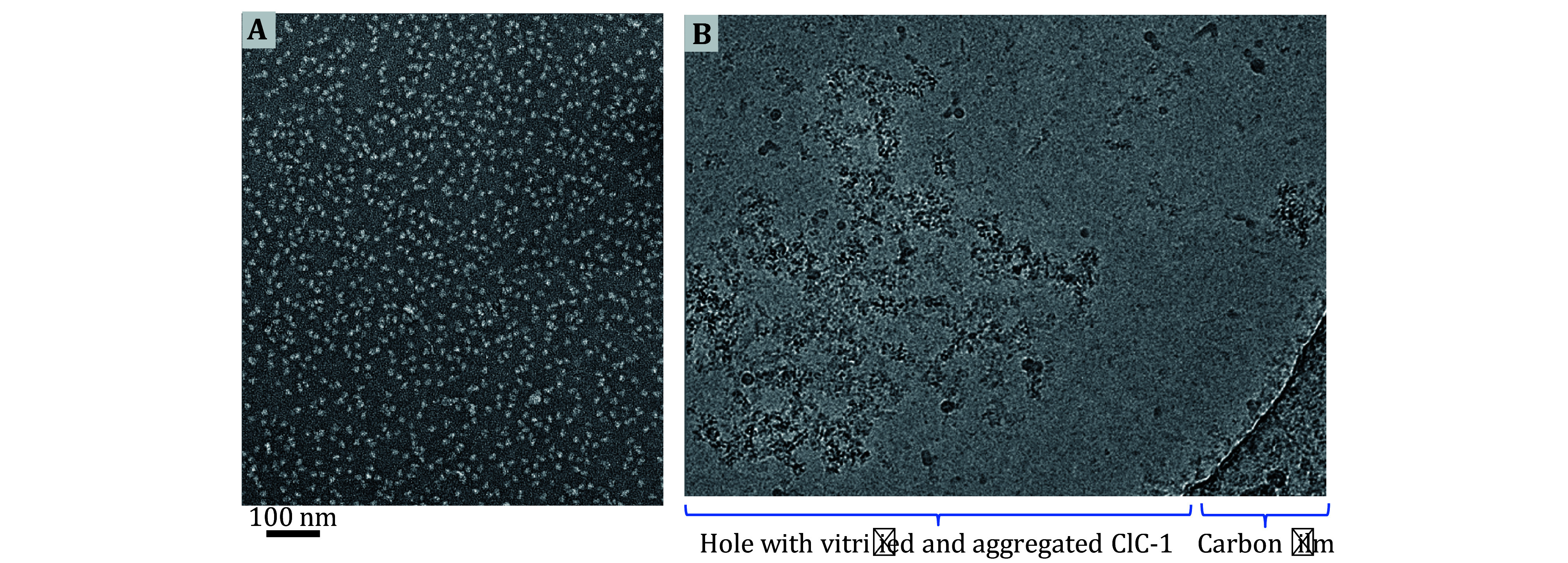
Example of the AWI adsorption problem in cryoEM. **A** Negative stain EM micrograph of purified human ClC-1 protein particles, showing intact particles and optimal particle distribution prior to vitrification. **B** CryoEM micrograph of frozen-hydrated human ClC-1 protein particles across a holey carbon film, showing the typical aggregation and deformation problem occurring at the AWI in the absence of adequate surfactant

Cryogenic electron microscopy (cryoEM) has become a tool of choice for determining the atomic structures of biological complexes. The general strategy in sample preparation involves three steps: First, the sample is placed on continuous carbon film, negatively stained, and evaluated by transmission electron microscopy (TEM) to optimize the purity and concentration of the sample. Second, a droplet of the optimized sample is applied to various cryoEM grids ( *e*. *g*., holey carbon or gold foil grids, graphene film grids, lacey carbon film grids, *etc*.) and blotted with filter paper to form a thin layer of liquid containing the sample. Third, the blotted grid is plunged into liquid ethane to flash-freeze the thin layer of liquid, embedding the sample in vitreous ice (Dubochet *et al.* 1988). After completing these steps, it is often observed that embedded particles of the sample ( *e*. *g*., Fig. 1), undergo aggregation and deformation at the air–water interface (AWI) (Taylor and Glaeser 2008), which can be surprising and disappointing. Cryogenic electron tomography (cryoET) revealed that a broad range of macromolecular complexes was adsorbed at the AWI in cryoEM grid holes when these standard grid preparation steps were used (Noble *et al.* 2018a). This problem, also known as the AWI adsorption phenomenon, causes an uneven distribution and preferred orientations of the particles, resulting in directional resolution anisotropy (Lyumkis 2019) — a major obstacle in high-resolution cryoEM structure determination (Bai *et al.* 2013; D'Imprima *et al.* 2019). This AWI adsorption phenomenon was reported several decades ago (Graham and Phillips 1979; MacRitchie 1985; Narsimhan and Uraizee 1992), affecting membrane and non-membrane proteins (Noble *et al.* 2018a). It has been proposed that this phenomenon results from the particles’ diffusion to the AWI due to their exposed hydrophobic regions (Glaeser and Han 2017). However, a complete understanding and elimination of this phenomenon remain elusive.

Despite the lack of understanding, numerous attempts have been made to develop practical methods to address the problem. For example, using a faster plunge device (such as Spotiton), whose elapsed time between applying the sample to grids and plunging into liquid ethane is in the order of 100 ms (much less than the traditional plunge of more than 1 s), can alleviate preferred orientations problem caused by the AWI phenomenon (Jain *et al.* 2012; Noble *et al.* 2018b). Also, using continuous thin layers of amorphous carbon (Williams and Glaeser 1972) or graphene (D'Imprima *et al.* 2019; Pantelic *et al.* 2011; Russo and Passmore 2014) has been found to mitigate the AWI adsorption problem. Although these methods may improve the cryoEM result, they still have limitations. For instance, using a fast nano-dispenser does not provide direct evidence of eliminating AWI absorption problems (Noble *et al.* 2018b). Using a thin layer of amorphous carbon as a support layer on cryoEM grids can adsorb and thus immobilize some particles, but it also adds background noise to cryoEM images (Russo and Passmore 2016). A more widely used method is to include surfactants or detergents in the sample solution to improve particle preservation on cryoEM grids, especially for membrane proteins. For example, zwitter-ionic detergent (CHAPSO) has been reported to reduce the AWI adsorption of bacterial RNA polymerase (Chen *et al.* 2019) and human erythrocyte catalase (Chen *et al.* 2022), as well as alleviate the preferred orientation problem. Regrettably, a specific surfactant may prove to be effective for one sample type but fail to yield similar results for others. Therefore, a deeper understanding of the mechanism of AWI is required to better utilize surfactants in cryoEM sample preparation.

In this study, we developed a theoretical framework to explain the AWI adsorption phenomenon in the context of surface energy. We also experimentally examined the effects of different surfactants on cryoEM sample preparation using the standard test sample, GroEL, using cryoET and cryoEM. According to this framework, particles migrate to AWI to decrease the overall area of the liquid exposed to the air, thereby minimizing the overall potential energy of the system. This framework rationalizes using surfactants to alleviate the AWI problem: They reduce the surface energy of an aqueous solution, diminishing the overall potential energy and minimizing the particles’ tendency to migrate towards AWI. To experimentally test the theory’s predictability, we used cryoET to visualize particle distribution across the sample depth on the cryoEM grid from one AWI to the other. Our cryoET results demonstrated the effectiveness of three surfactants (NP40, DDM, and fluorinated fos-choline 8 (FFC8)) against the AWI adsorption problem. We also conducted single-particle cryoEM and 3D reconstruction with GroEL to demonstrate that these surfactants did not adversely affect the protein structures at near-atomic resolution. We further showed the effectiveness of FFC8 in drastically improving particle distribution and generating a near-atomic resolution structure of a more challenging membrane protein, the ClC-1 channel.

## RESULTS

### Theoretical formulation of the air−water interface problem in cryoEM

All matter, except for ideal gases, is held together by molecules that exhibit varying degrees of attraction to one another. In the bulk of matter, the intermolecular forces equilibrate each other. However, at the surface of matter, molecules are not fully surrounded by their neighbors, which results in a net inward force pointing into the bulk (Hauner *et al.* 2017). This net inward force increases as the exposed surface area of matter increases. To achieve equilibrium, work must be done to counterbalance this net inward force. The greater the exposed surface area of matter, the greater the work to counterbalance the net inward force. The ratio between the work and surface area, or work per unit surface area, can be represented by $ \gamma $ :

1 $ \gamma =\frac{\Delta Work}{\Delta Area}=\frac{Joules}{{m}^{2}}\;, $

which is also known simply as surface energy in the field of surface science and fluid dynamics. Surface energy is due to the attraction of electrical charges around the molecules. Surface energy is then defined as the work required to disrupt these intermolecular attractions and pull apart molecules to a certain extent of area.

As surface energy is applied to the matter to stretch its surface, the surface of the matter counteracts the surface increase through the tangential tension force. This isotropic surface stress associated with deformation is called surface tension. The terms “surface tension” and “surface energy” refer to the same unit, as demonstrated in the following analysis, although they may not necessarily have the same value:

2 \begin{equation*}\begin{split} Surface\;\;energy\left(\gamma \right)&=\frac{\Delta Work}{\Delta Area}=\frac{Joules}{{m}^{2}} =\frac{Newton\;\times\;m}{{m}^{2}} \\& =\frac{Newton}{m} =\frac{\Delta Tangential\;tension\;force}{\Delta Length}\\&= Surface\;tension \;. \end{split}\end{equation*}

For liquid, the two values are the same because as the surface deforms, the molecules in the bulk can freely move to the deforming surface and the intermolecular distance at the surface does not change. As such, two terms are often used interchangeably for liquids. However, for a solid, the surface molecules remain constant, so the work required for surface deformation is dependent on the intermolecular distance. As a result, this work is not the same as the work required for creating a new surface (Mondal *et al.* 2015). As our paper deals with both solid and liquid surfaces, only the term “surface energy” will be used henceforth for simplicity.

The surface energy of water is one of the highest (72 J/m ^2^ at room temperature and atmospheric pressure) because of a strong hydrogen bond between water molecules (Hauner *et al.* 2017). Interestingly, in a cryoEM grid, the solid particles are primarily drawn to the liquid surface, despite the high surface energy there. Such behavior is also known as the air water–interface (AWI) adsorption phenomenon. To explain this phenomenon, which at a glance seems to defy the laws of physics, we provide time-lapsed schematics of three main stages and the mathematical equation that accounts for the area of all interfaces and the respective surface energies. The equation calculates the overall potential energy for each stage, allowing for direct comparison among the stages.

**Figure 2:**
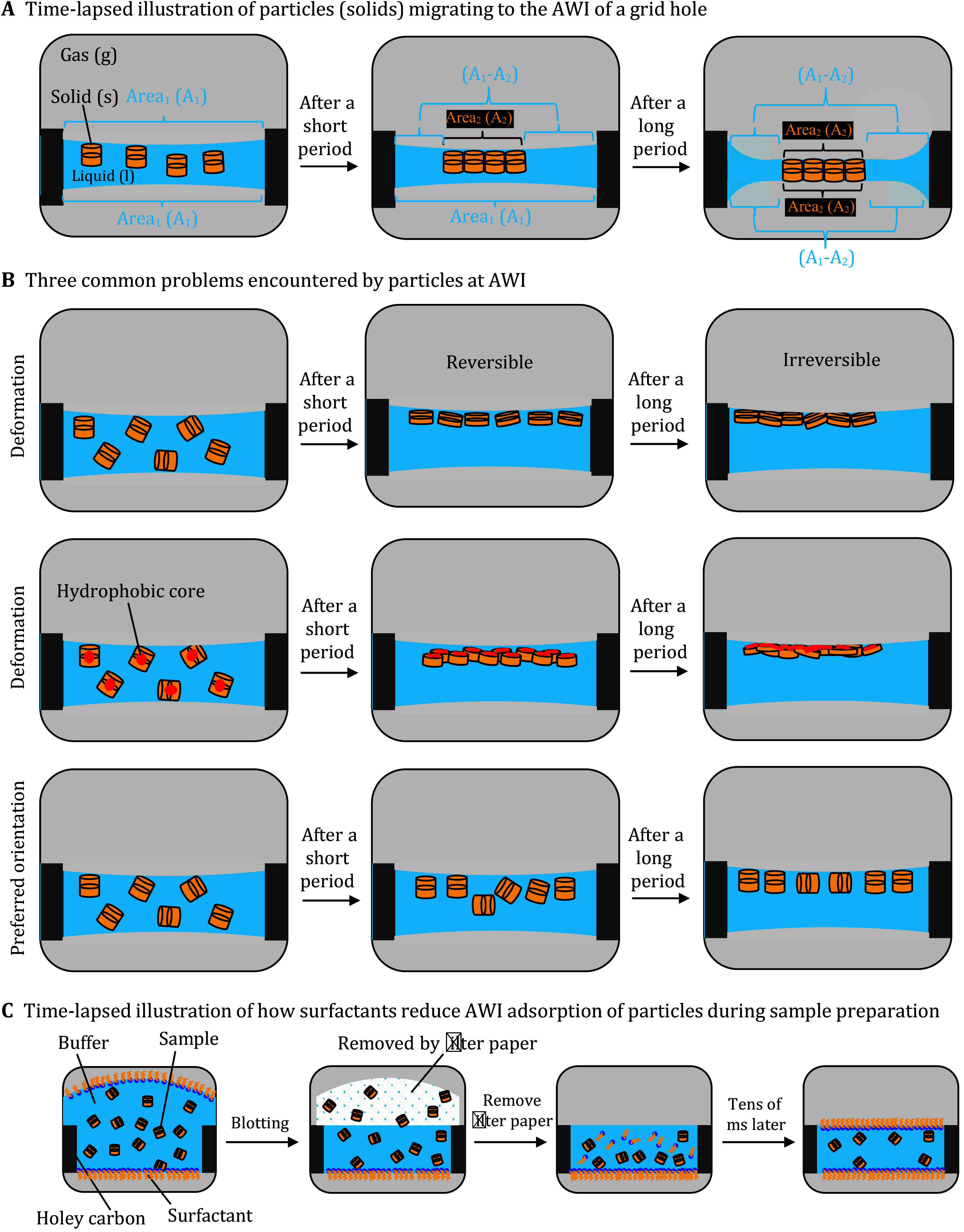


In the first stage immediately after the sample is applied to a grid, the particles are within the sample’s bulk volume, suspended across a grid hole away from the AWI ( Fig. 2A, left). Since the surface energy ( $ \gamma $ ) is defined as the work required to build a unit area, the work (in *Joules*) needed to build a surface is the product of $ \gamma \left(\mathrm{i}\mathrm{n}\;\dfrac{Joules}{area}\right) $ and the area created. Based on Navier-Stokes equations, which describe the motion of viscous fluid substances (Temam 2001), the overall potential energy ( *Ω* in *Joules*) for all the surfaces along the vertical axis can be calculated as the following:

**Figure 2:** Side-view schematics of a hole of cryoEM grid, highlighting particle behavior and distribution in relation to AWI. **A** Time-lapsed illustration of AWI adsorption of particles in the buffer. First, particles stay in the bulk volume (Left panel). A1 denotes the surface area of the buffer layer at the AWI, *i*. *e*., the size of the grid hole. There are two A1’s, one at the top and one at the bottom. After a short period, particles adsorb to one of the two AWIs, achieving a more favorable energy state (one-side AWI adsorption) (Center panel). A2 denotes the overall sum of the area of particles’ surfaces exposed to the air, upon migrating to the AWI. At the top AWI, the overall A1 decreases as the particles occupy the surface, unlike the unoccupied A1 at the bottom AWI. Eventually, particles adsorb to the remaining AWI as the buffer layer thins, achieving the most favorable energy state (two-side AWI adsorption) (Right panel). At the bottom AWI, the overall A1 also decreases due to the same particles occupying that surface. **B** Three common problems encountered by the sample particles at AWI, namely, particle deformation, denaturation, and preferred orientation. **C** Time-lapsed illustration of how surfactants reduce or even eliminate AWI adsorption of particles during sample preparation. First, the sample-surfactant mixture is loaded from the top side, denoted by the bulge (first panel). Surfactants migrate more quickly than the sample particles (see explanation in the result section) to occupy the two AWIs, thereby blocking sample particles from migrating to the surfaces. Upon blotting, some particles and surfactants are removed by the filter paper (second panel), exposing a second surface at the blotted side (third panel), which would also be occupied by the surfactant molecules, preventing the remaining sample particles from migrating to the free surface (last panel)

3 $ {\Omega }_{\mathrm{b}\mathrm{u}\mathrm{l}\mathrm{k}}=2{A}_{1}{\gamma }_{\mathrm{l}\mathrm{g}} + 2{A}_{2}{\gamma }_{\mathrm{l}\mathrm{s}}\;, $

where $ {\gamma }_{\mathrm{l}\mathrm{g}} $ is the surface energy generated by the buffer solution (liquid) interfacing with the surrounding air (gas); *A*_1_ is the liquid surface area, which is essentially equivalent to the size of the grid hole; $ {\gamma }_{\mathrm{l}\mathrm{s}} $ is the surface energy at the top and bottom surfaces of the particle (solid) interfacing with the buffer solution (liquid); *A*_2_ is the top or bottom surface area of the solids, which is essentially half of the combined areas of all particles.

In the second stage, a particle adsorbs to one of the AWIs, which we call the single adsorption state ( Fig. 2A, center). The overall potential energy of the single adsorption state can be calculated as,

4 \begin{equation*}\begin{split} \Omega_{\mathrm{s}\mathrm{i}\mathrm{n}\mathrm{g}\mathrm{l}\mathrm{e}\mathrm{ }\mathrm{a}\mathrm{d}\mathrm{s}\mathrm{o}\mathrm{r}\mathrm{p}\mathrm{t}\mathrm{i}\mathrm{o}\mathrm{n}}& ={A}_{1}{\gamma }_{\mathrm{l}\mathrm{g}} + {A}_{2}{\gamma }_{\mathrm{l}\mathrm{s}} + {A}_{2}{\gamma }_{\mathrm{s}\mathrm{g}} + \left({A}_{1}-{A}_{2}\right){\gamma }_{\mathrm{l}\mathrm{g}} \\& ={A}_{1}{\gamma }_{\mathrm{l}\mathrm{g}} + {A}_{2}{\gamma }_{\mathrm{l}\mathrm{s}} + {A}_{2}{\gamma }_{\mathrm{s}\mathrm{g}} + {A}_{1}{\gamma }_{\mathrm{l}\mathrm{g}}-{A}_{2}{\gamma }_{\mathrm{l}\mathrm{g}} \\& =2{A}_{1}{\gamma }_{\mathrm{l}\mathrm{g}} + {A}_{2}{\gamma }_{\mathrm{l}\mathrm{s}} + {A}_{2}{\gamma }_{\mathrm{s}\mathrm{g}}-{A}_{2}{\gamma }_{\mathrm{l}\mathrm{g}}\;, \end{split}\end{equation*}

where a new surface energy, $ {\gamma }_{\mathrm{s}\mathrm{g}} $ , is generated by the particle’s top surface. As the particles occupy the top surface, the working surface area for $ {\gamma }_{\mathrm{l}\mathrm{g}} $ decreases, resulting in a reduction of the total work exerted by the surface energy. The change in the overall potential energy of $ {\Omega }_{\mathrm{b}\mathrm{u}\mathrm{l}\mathrm{k}} $ from $ {\Omega }_{\mathrm{s}\mathrm{i}\mathrm{n}\mathrm{g}\mathrm{l}\mathrm{e}\mathrm{ }\mathrm{a}\mathrm{d}\mathrm{s}\mathrm{o}\mathrm{r}\mathrm{p}\mathrm{t}\mathrm{i}\mathrm{o}\mathrm{n}} $ to $ {\Omega }_{\mathrm{b}\mathrm{u}\mathrm{l}\mathrm{k}} $ can be expressed as,

5 $ {\Omega }_{\mathrm{b}\mathrm{u}\mathrm{l}\mathrm{k}}-{\Omega }_{\mathrm{s}\mathrm{i}\mathrm{n}\mathrm{g}\mathrm{l}\mathrm{e}\mathrm{ }\mathrm{a}\mathrm{d}\mathrm{s}\mathrm{o}\mathrm{r}\mathrm{p}\mathrm{t}\mathrm{i}\mathrm{o}\mathrm{n}}={A}_{2}{\gamma }_{\mathrm{l}\mathrm{s}} + {A}_{2}{\gamma }_{\mathrm{s}\mathrm{g}}-{A}_{2}{\gamma }_{\mathrm{l}\mathrm{g}} \;,$

which can be further simplified using Young’s equation,

6 $ {\gamma }_{\mathrm{s}\mathrm{g}}={\gamma }_{\mathrm{s}\mathrm{l}} + {\gamma }_{\mathrm{l}\mathrm{g}}\mathrm{c}\mathrm{o}\mathrm{s}\theta \;,$

where *θ* is the equilibrium contact angle that the liquid forms with the solid (Shuttleworth 1950; Hiemenz and Rajagopalan 1997).

In the case of single adsorption, the contact angle between the liquid and solid is 180°, simplifying the equation to

7 $ {\gamma }_{\mathrm{s}\mathrm{g}}={\gamma }_{\mathrm{s}\mathrm{l}}-{\gamma }_{\mathrm{l}\mathrm{g}}\;. $

Using this relationship, Eq. 5 is simplified to

8 $ {\Omega }_{\mathrm{b}\mathrm{u}\mathrm{l}\mathrm{k}}-{\Omega }_{\mathrm{s}\mathrm{i}\mathrm{n}\mathrm{g}\mathrm{l}\mathrm{e}\mathrm{ }\mathrm{a}\mathrm{d}\mathrm{s}\mathrm{o}\mathrm{r}\mathrm{p}\mathrm{t}\mathrm{i}\mathrm{o}\mathrm{n}}=2{A}_{2}{\gamma }_{\mathrm{s}\mathrm{g}} \;,$

indicating a decrease in the overall potential energy as the system transitions from the bulk state to the single adsorption state.

Because the particle adsorption to the surface leads to a more energetically favorable state, the remaining particle side will likely follow the same path, leading to a double adsorption state ( Fig. 2A, right). The overall potential energy of the surface of double adsorption can be expressed as

9 $ {\Omega }_{\mathrm{d}\mathrm{o}\mathrm{u}\mathrm{b}\mathrm{l}\mathrm{e}\mathrm{ }\mathrm{a}\mathrm{d}\mathrm{s}\mathrm{o}\mathrm{r}\mathrm{p}\mathrm{t}\mathrm{i}\mathrm{o}\mathrm{n}}=2{A}_{1}{\gamma }_{\mathrm{l}\mathrm{g}} + 2{A}_{2}{\gamma }_{\mathrm{s}\mathrm{g}}-2{A}_{2}{\gamma }_{\mathrm{l}\mathrm{g}}\;. $

Comparing the potential energy between $ {\Omega }_{\mathrm{s}\mathrm{i}\mathrm{n}\mathrm{g}\mathrm{l}\mathrm{e}\mathrm{ }\mathrm{a}\mathrm{d}\mathrm{s}\mathrm{o}\mathrm{r}\mathrm{p}\mathrm{t}\mathrm{i}\mathrm{o}\mathrm{n}} $ and $ {\Omega }_{\mathrm{d}\mathrm{o}\mathrm{u}\mathrm{b}\mathrm{l}\mathrm{e}\mathrm{ }\mathrm{a}\mathrm{d}\mathrm{s}\mathrm{o}\mathrm{r}\mathrm{p}\mathrm{t}\mathrm{i}\mathrm{o}\mathrm{n}} $ ,

10 $ {\Omega }_{\mathrm{s}\mathrm{i}\mathrm{n}\mathrm{g}\mathrm{l}\mathrm{e}\mathrm{ }\mathrm{a}\mathrm{d}\mathrm{s}\mathrm{o}\mathrm{r}\mathrm{p}\mathrm{t}\mathrm{i}\mathrm{o}\mathrm{n}}-\Omega _{\mathrm{d}\mathrm{o}\mathrm{u}\mathrm{b}\mathrm{l}\mathrm{e}\mathrm{ }\mathrm{a}\mathrm{d}\mathrm{s}\mathrm{o}\mathrm{r}\mathrm{p}\mathrm{t}\mathrm{i}\mathrm{o}\mathrm{n}}={A}_{2}{\gamma }_{\mathrm{l}\mathrm{s}}-2{A}_{2}{\gamma }_{\mathrm{s}\mathrm{g}} + {A}_{2}{\gamma }_{\mathrm{l}\mathrm{g}}\;. $

Using the relationship established in Eq. 7, the above difference is simplified to

11 $ {\Omega }_{\mathrm{s}\mathrm{i}\mathrm{n}\mathrm{g}\mathrm{l}\mathrm{e}\mathrm{ }\mathrm{a}\mathrm{d}\mathrm{s}\mathrm{o}\mathrm{r}\mathrm{p}\mathrm{t}\mathrm{i}\mathrm{o}\mathrm{n}}-\Omega _{\mathrm{d}\mathrm{o}\mathrm{u}\mathrm{b}\mathrm{l}\mathrm{e}\mathrm{ }\mathrm{a}\mathrm{d}\mathrm{s}\mathrm{o}\mathrm{r}\mathrm{p}\mathrm{t}\mathrm{i}\mathrm{o}\mathrm{n}}=2{A}_{2}{\gamma }_{\mathrm{l}\mathrm{g}}\;, $

indicating a decrease in the overall potential energy as the system transitions from the single adsorption state to the double adsorption state. This explains why the ice thickness of a properly blotted cryoEM grid tends to be the same as the particle thickness. Indeed, the adsorption phenomenon in AWI, despite the particles’ migration to the surface ( *i*. *e*., a high surface energy region), is logical as it generates the most energetically favorable state.

### How is sample aggregation related to AWI

As commonly observed in electron microscopy, some particles aggregate at the AWI. Protein aggregation is the aftermath of protein denaturation that causes the exposed hydrophobic cores of the individual proteins to combine together (Koepf *et al.* 2017), the event prevalent at the AWI (D'Imprima *et al.* 2019; Wiesbauer *et al.* 2013). To explain the connection between AWI and protein denaturation, we adopted a coarse-grained structure-based model that describes the force exerted on the particles at the AWI (Cieplak *et al.* 2014),

12 $ {F}_{i}^{wa}={q}_{i}A\frac{\mathrm{e}\mathrm{x}\mathrm{p}\left({-{z}_{i}}^{2}/{2W}^{\;2}\right)}{\sqrt{2{\pi} }W} \;.$

In this model, *A* refers to the amplitude of the depth of the potential in the effective contact interaction between two residues, A higher value of *A* indicates a greater degree of pinning of a molecule to the water surface; *W* is the width of the interface; $ {q}_{i} $ is the hydropathy index of amino acid, ranging from ‒4.5 for the polar arginine to 4.5 for the most hydrophobic isoleucine. The AWI is centered at *z* = 0, where *Δz* indicates protein deformation. This phenomenological model suggests that the force exerted at the AWI denatures protein, leading to the readjustment of the protein orientation to balance the hydropathy-related forces. By comparing proteins with varying degrees of hydrophilicity, others have demonstrated that the AWI adsorption occurrence is proportional to both the protein’s hydrophobic residues and the protein deformation events (Zhao *et al.* 2017). To equilibrate the hydropathy-related forces, the hydrophilic and hydrophobic residues of the protein pull toward the bulk and AWI, respectively, resulting in protein deformation at the AWI.

Since there are charged molecules in the sample, we also considered electrostatic forces. Toward this end, we explored the concept of Debye-Hückel length (Kirby 2010), which measures how far the charge-carrying species’ net electrostatic effect persists in a solution. This length is defined as

13 $ \lambda _{D}={\left(\frac{\varepsilon {k}_{\mathrm{B}}T}{{\displaystyle\sum }_{j}^{N}={{1}^{n}}_{j}^{o}{q}_{j}^{2}}\right)}^{\tfrac{1}{2}} \;,$

where $ \epsilon $ is the relative static permittivity of solvent, $ {k}_{B} $ is Boltzmann’s constant, $ T $ is the temperature, $ {n}_{j} $ is the mean concentration of charges of the species *j*, and $ {q}_{j} $ is the charge of the species *j*. The Debye length in phosphate-buffered saline (PBS) at room temperature, a commonly used solution in biology and cryoEM research, is 0.7 nm (Chu *et al.* 2017), which is approximately the diameter of α helix secondary structure in proteins and significantly smaller than the size of folded proteins and their domains. As such, we concluded that the electrostatic force is negligible when considering molecular interactions at the AWI.

Lastly, we considered protein denaturation in relation to energy. In the energy landscape, unfolding events involve a series of small, destabilizing (uphill) steps, with small bumps (activation barriers) and dips (local minima), which are driven by the constant, reversible “sub-globally cooperative unfolding/refolding” events (Glaeser and Han 2017; Maity *et al.* 2005) that occur at the molecular level. A native protein initially adsorbs to the AWI to reach a more energetically favorable state. After reaching a transitional state, *i*. *e*., the peak of the energy barrier, the protein spontaneously proceeds downhill and unfolds (Glaeser and Han 2017). Hydrogens within the native protein can exchange with solvent as the native hydrogen bonds are transiently broken. The cytochrome *c* (Cyt *c*) results showed five sub-globally cooperative units, called foldons, that fold and unfold stepwise, whose amide hydrogen exchange is demonstrated by small local structural fluctuations that break one hydrogen bond at a time (Maity *et al.* 2005). Results from the unfolding of RNase H showed that it begins with the local fluctuations of the least stable protons on the side of the helix that is more solvent-exposed before the global unfolding. Both events place amide groups in proton exchange-competent forms in the solvent (Chamberlain *et al.* 1996). These transient events expose additional hydrophobic groups of the sample, causing them to adsorb to the AWI irreversibly. With no activation barrier at the surface, a spontaneous, non-reversible “catastrophic” denaturation occurs, leading to the unfolded proteins. Exposed internal regions of these unfolded proteins are typically hydrophobic, which promotes sample aggregation, a hallmark of the AWI problem.

### Surfactant application in cryoEM sample preparation alleviates the AWI adsorption problem by shifting the equilibrium

A surfactant is an amphipathic surface-active molecule, with its polar head in the water and non-polar tail exposed to the air. Surfactants have been widely used in cryoEM to alleviate the AWI adsorption phenomenon. Our formulation (Eq. 7) predicts that all particles would migrate to the AWI, as that would lower the potential energy of the system ( *i*. *e.*, make it more negative *Ω*). It also predicts ways to solve or alleviate the AWI problem: by eliminating such aqueous surfaces of the system, or if impossible, by eliminating sample particles’ access to the surface, and decreasing the surface energy. For the latter, surfactants can lower the surface energy ( *i*. *e.*, $ {\gamma }_{\mathrm{l}\mathrm{g}} $ above), thus rationalizing the empirical use of surfactants in alleviating the AWI problem (Liao and Zatz 1979). Surfactants reduce the surface energy of the aqueous solution because when surfactant molecules populate the water surface, they replace the existing hydrogen bonds between water molecules. As a result, surface energy ( $ {\gamma }_{\mathrm{l}\mathrm{g}} $ ) gradually decreases until reaching the minimum at the surfactant’s critical micelle concentration (CMC), the concentration above which surfactants form micelles (Liao and Zatz 1979). Importantly, thanks to their hydrodynamic radius, surfactants will migrate faster to the surface than the sample molecules, which typically are much larger macromolecular complexes, protecting the sample molecules from denaturation. In the extreme scenario, wherein all sites on the AWI are occupied by the surfactant molecules, the exposed surfaces accessible to sample molecules are entirely eliminated. This situation leads to an ideal condition where an aqueous surface is present for the macromolecular complexes to be visualized using cryoEM.

### Experimental confirmation of the air−water interface adsorption problem

CryoEM sample is embedded in vitreous ice, which helps retain near-native high-resolution features of the sample. The aqueous buffer, which becomes vitreous ice through flash freezing, serves as a double-edged sword in that it is crucial for providing a “near-native” environment for the sample, but its existence introduces AWI adsorption phenomenon, causing denaturation, deformation, and preferred orientation of the sample. To address these potential problems in cryoEM sample preparation, we evaluated the behavior of a widely tested non-membrane protein, GroEL. We used cryoET to reveal the 3D structure of the embedding ice in the hole of the holey carbon grids to assess the GroEL distribution throughout the grid thickness.

**Figure 3:**
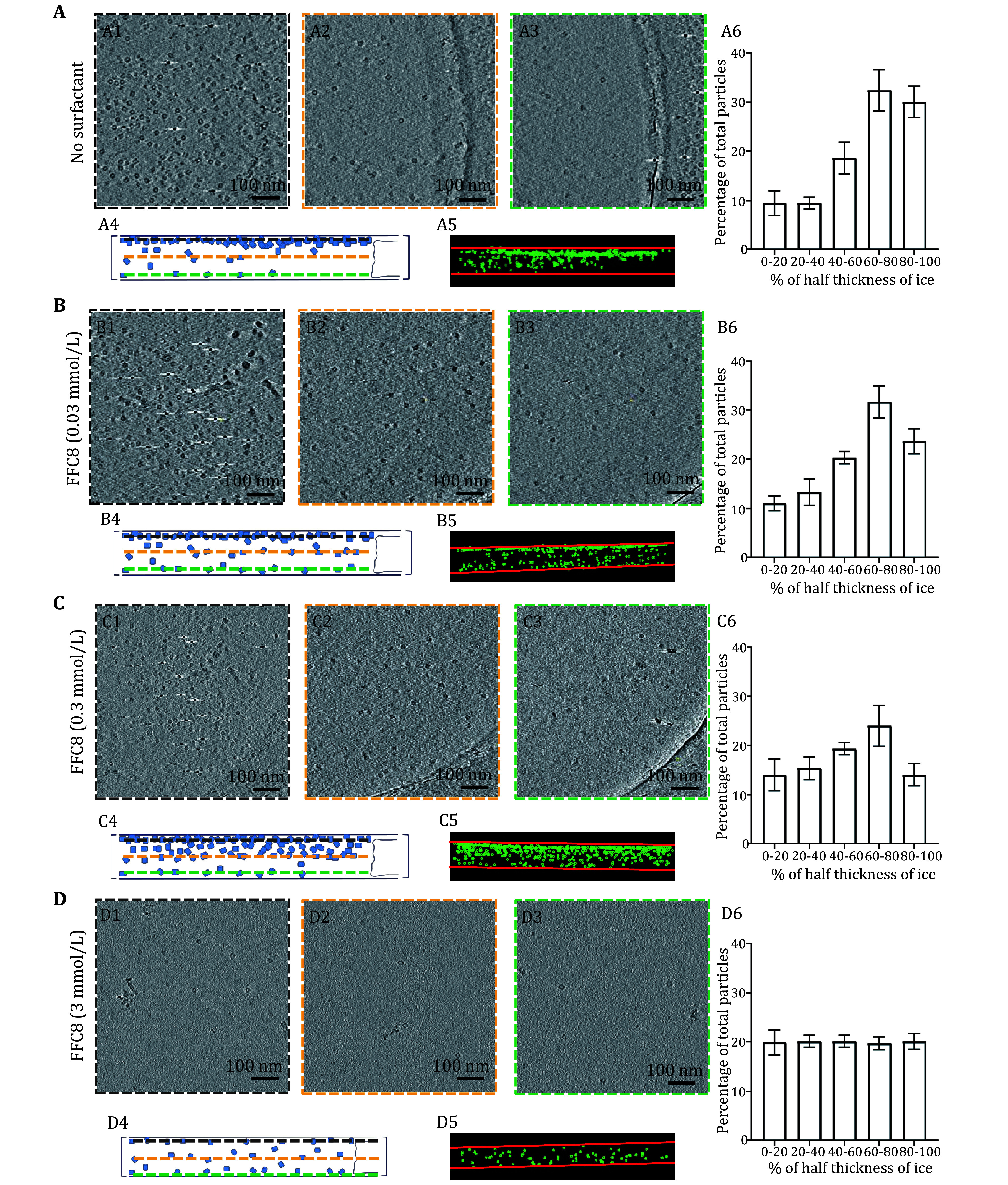


After generating 3D reconstructions using the IMOD package (Kremer *et al.* 1996), we could assess the particle distribution throughout the ice thickness, as shown in Fig. 3. The slice of tomograms (about 5-nm thick) was extracted from two AWIs and the middle region of the ice. Without surfactant ( Figs. 3A1–3A3), most particles were observed at the AWIs, indicating that GroEL particles, like other complexes, are prone to AWI adsorption problems. More specifically, most particles were on the opposite surface ( Fig. 3A1, Top slice) of the sample-applied surface ( Fig. 3A3, bottom slice).

**Figure 3:** GroEL particle distribution in the vitrified sample with different concentrations of FFC8. **A**– **D** Each alphabetized panel contains six numericized sub-sections ( *e*. *g*., Panels A1 through A6 or D1 through D6), organized in the same manner for all panels. Panels A1 through A6 pertain to no-surfactant conditions, and Panels B through D pertain to different FFC8 concentrations as indicated. The first three sub-sections (1–3) show the top, center, and bottom slice of the tomogram from cryoET, respectively, viewed from the top of the grid. Sub-section 4 shows the schematic of Sub-sections 1–3, viewed from the side of the grid. The dashed border (black, orange, and green) in Sub-sections 1–3 corresponds to the dashed horizontal line (black, orange, and green) in Sub-section 4. Sub-section 5 shows the actual particle distribution of the tomogram, viewed from the side, with green dots representing GroEL particles and red horizontal lines representing the AWIs. Sub-section 6 shows the histogram of particle distribution across the entire ice thickness. The *X*-axis is divided into ranges based on the magnitude of deviation (represented in %) from the center of the ice ( *e*. *g*., 0% indicating no deviation from the center of the ice and 100% representing the maximum deviation from the center, *i*. *e*., either side of AWI). The *Y*-axis represents the number of particles (represented as the percentage of the total population) residing in each ice region

Next, we analyzed the side view from our reconstructed tomogram to better understand the particle distribution ( Figs. 3A4 and 3A5). The degree of deviation away from the middle was expressed in percentage. The middle plane and the surfaces were set to 0 and 100%, respectively ( Fig. 3A6). Thirty percent of the particles fell within the 80% to 100% range, while 33% were in the 60% to 80% range. Taken together, our results indicated that GroEL suffers from the AWI adsorption problem.

### Different surfactants at varying concentrations can alter particle distribution in ice

Upon confirming the AWI absorption problem in GroEL, we experimentally validated the effectiveness of surfactants. Our first surfactant of choice was FFC8, which can help form a thinner buffer layer for cryoEM samples (Glaeser 2018; Hughes *et al.* 2018; Johnson and Chen 2017) than the non-fluorinated counterparts. Also, FFC8 has a relatively high CMC (3.0 mmol/L) compared to many other surfactants, whose CMCs are generally less than 1 mmol/L (Inácio *et al.* 2011), alleviating the micelle-induced perturbation of image contrast. As a result, FFC8 tolerates a broader range of applicable concentrations. Lastly, FFC8 contains a polar tail that is both hydrophobic and oleophobic, reducing the likelihood of biomolecule denaturation (Park *et al.* 2007).

We prepared three samples of 2 mg/mL GroEL, each with a different concentration of FFC8 (1 × CMC, 1:10 CMC, and 1:100 CMC FFC8) added immediately prior to freezing the grid, and cryoET tilt series were collected and reconstructed. The number of particles in three tomogram slices (about 5-nm thick), including two slices for AWI layers and one slice for the middle layer, was compared among the samples in the above-mentioned concentrations ( Figs. 3B1–3B3, 3C1–3C3, 3D1–3D3). As the FFC8 concentration increased, the distribution bias toward the AWI decreased ( Figs. 3B4–3B6, 3C4–3C6). At CMC, particles were evenly distributed throughout the ice thickness ( Figs. 3D4–3D6). The overall particle number significantly decreased at CMC, which indicated a more even particle distribution, as shown in our calculation from the previous section. Our findings are in line with the previous study that highlighted surfactants’ effectiveness in relieving the oversaturation of the field of view (Roh *et al.* 2017).

**Figure 4:**
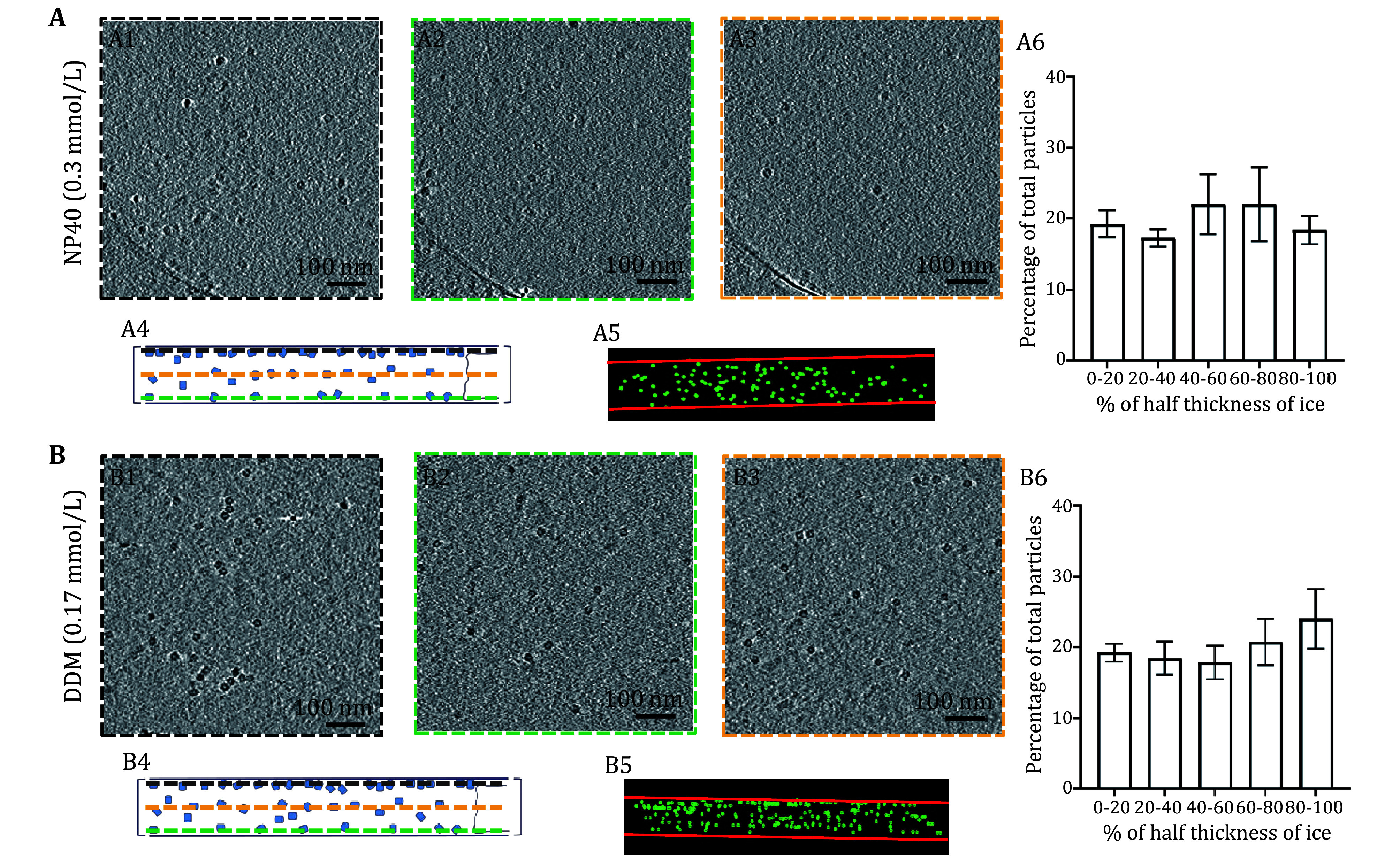
GroEL particle distributions in vitrified samples with different surfactants at their respective CMC. **A**– **B** Each alphabetized panel contains six numericized sub-sections ( *e*. *g*., Panels A1 through A6 or B1 through B6), organized in the same fashion for all panels. Panels A1 through A6 pertain to NP40 at CMC condition and B1 through B6 pertain to DDM at CMC condition as indicated. The first three sub-sections (1–3) show the top, center, and bottom slice of the tomogram from cryoET, respectively, viewed from the top of the grid. Sub-section 4 shows the schematic of Sub-sections 1–3, viewed from the side of the grid. The dashed border (black, orange, and green) in Sub-sections 1–3 corresponds to the dashed horizontal line (black, orange, and green) in Sub-section 4. Sub-section 5 shows the actual particle distribution of the tomogram, viewed from the side, with green dots representing GroEL particles and red horizontal lines representing the AWIs. Sub-section 6 shows the histogram of particle distribution across the entire ice thickness. The *X*-axis is divided into ranges based on the magnitude of deviation (represented in %) from the center of the ice ( *e*. *g*., 0% indicating no deviation from the center of the ice and 100% representing the maximum deviation from the center, *i*. *e*., either side of AWI). The *Y*-axis shows the number of particles (represented as the percentage of the total population) residing in each ice region

Aside from the fluorinated surfactant, we also tested non-ionic surfactants such as dodecyl maltoside (DDM) and NP-40, commonly used for solubilizing membrane proteins for cryoEM studies (Vinothkumar 2015). Like FFC8 at CMC, the addition of NP-40 at CMC (0.3 mmol/L) and DDM at CMC (0.17 mmol/L) resulted in even particle distribution of GroEL ( Fig. 4). These results indicated that DDM and NP40, surfactants of choice for membrane proteins, can also be effective on cytosolic proteins, such as GroEL, suggesting a broad spectrum of surfactant efficacy in improving the particle distribution. In summary, using reconstructed cryoET tomograms, we determined that the particle distribution in ice is directly related to surfactant concentration, reaching near elimination of AWI absorption phenomenon at CMC.

### Impact of commonly used surfactants on high-resolution cryoEM

**Figure 5:**
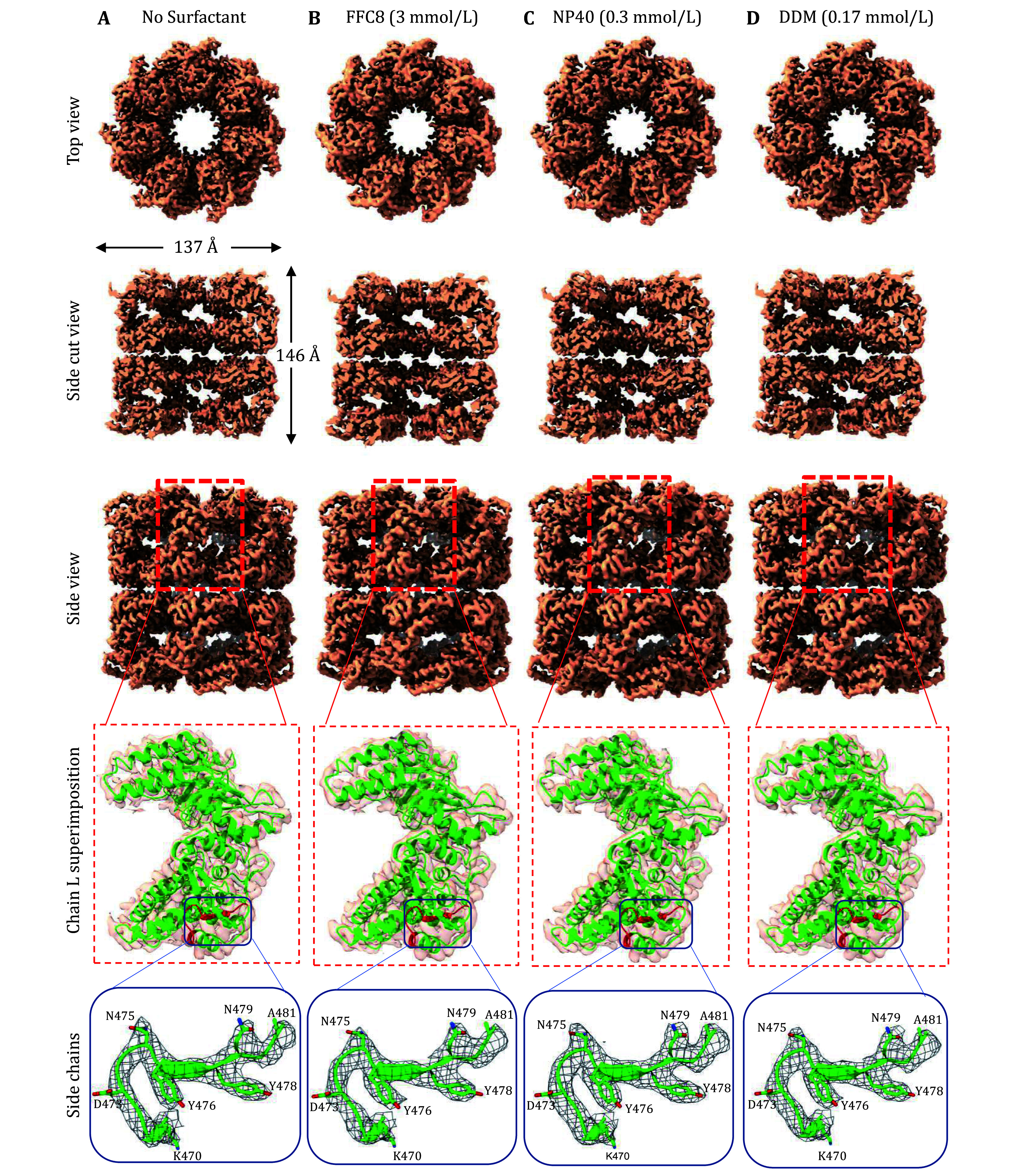
Near-atomic cryoEM reconstructions of GroEL, demonstrating no structural damage introduced by the surfactants at the indicated concentrations — the proposed solution to the AWI problem. **A**– **D** Various surface views of the cryoEM reconstructions of GroEL in different buffer conditions, in the absence of any surfactant at 3.3 Å resolution ( **A**), and the presence of 3.0 mmol/L FFC8 at 3.5 Å resolution ( **B**), 0.3 mmol/L NP40 at 3.3 Å resolution ( **C**), and 0.17 mmol/L DDM at 3.5 Å resolution ( **D**). The fourth row displays the semi-transparent zoom-in view of the boxed region in the third row, depicting a ribbon diagram of chain L of the X-ray crystal structure (Wang and Chen 2003) (PDB: 1MNF, shown in green ribbon representation) fitted. The fifth row shows the zoom-in view of the boxed region in the fourth row. The structure colored in red within the box (amino acid residues 470 to 481) is now shown with the amino-acid residues and the cryoEM densities are displayed as wireframes

Surfactant at high concentration is commonly used in molecular biology research to denature protein, namely, sodium dodecyl sulfate in SDS-PAGE. However, the denaturing ability of surfactants is undesirable in structural studies. To test whether the surfactants harm the protein structure, we performed a single-particle cryoEM analysis to assess the structural integrity of GroEL in the presence of surfactants. Approximately 500 to 700 micrographs were collected for the conditions above-mentioned (at CMC for conditions with surfactants). Subsequently, 3D reconstructions derived from the extracted particles were compared ( Fig. 5). 3D classification into four classes generated one “good” class average for all conditions, which was selected for the 3D refinement. The number of particles was normalized to 20,936 for all conditions. We were able to reach 3.3, 3.5, 3.5, and 3.3 Å resolution for no-surfactant, DDM, FFC8, and NP40 conditions, respectively. Chain L of the X-ray crystal structure (PDB: 1MNF (Wang and Chen 2003)) was fitted into the corresponding cryoEM density of all reconstructions to validate the structural integrity ( Fig. 5) at the level of amino-acid side chains (bottom row of Fig. 5). Overall, these surfactants at CMC did not induce structural damage of the particles.

**Figure 6:**
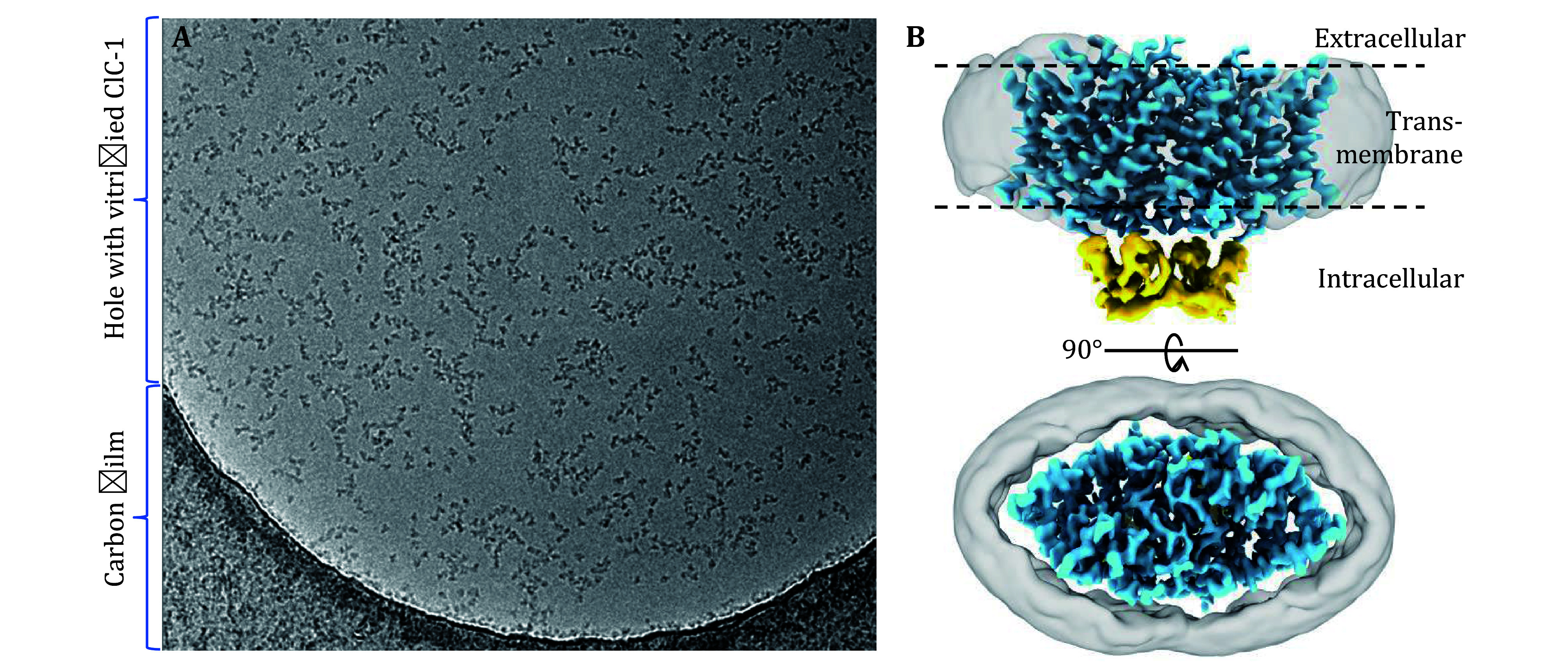
Application to real-world AWI adsorption problem. **A** A representative micrograph of single-particle cryoEM of purified human ClC-1 protein after applying FFC8 (at CMC, 3 mmol/L). As can be compared with Fig. 1, adding surfactant effectively solved the protein aggregation and deformation problem. **B** Two orthogonal shaded surface views of the resultant cryoEM map at an overall resolution of 3.6 Å, enabling *de novo* atomic modeling of the membrane domain (blue) of the complex (Wang *et al.* 2019). The intracellular domain is shown in yellow, and the detergent belt is in semi-transparent gray

Having demonstrated that surfactants can prevent AWI adsorption while maintaining the structural integrity of a stable, soluble protein, GroEL, we investigated whether this approach could provide a solution to a more challenging, real-world problem, namely, ClC-1, as shown in Fig. 1. ClC-1 is a membrane channel essential for maintaining Cl permeability across the plasma membrane of skeletal muscle fibers, accounting for 80% of the resting membrane conductance in humans (Stauber *et al.* 2012). Initially, the common phenomena that arose when the transitioning samples from the negative-stain EM grid to the cryoEM grid, such as particle aggregation and deformation, were also observed in ClC-1 ( Fig. 1). The application of FFC8 at CMC (3 mmol/L) prior to vitrification, led to mono-dispersion of ClC-1 particles ( Fig. 6A), suggesting that the samples no longer migrated to the AWI, thus preventing deformation and subsequent aggregation. This step was essential in producing good quality particles and the eventual 3.6 Å overall resolution cryoEM density map of the transmembrane domain, enabling *de novo* atomic model building (Wang *et al.* 2019) ( Fig. 6B). Taken together, surfactants application is an effective and practical solution to alleviating AWI adsorption problem in cryoEM.

## DISCUSSION

As cryoEM becomes widely recognized in the structural biology community as a tool of choice for determining atomic structures of biological complexes, the cases of these complexes encountering problems of preferential orientation, aggregation, or mysterious “disappearance” on cryoEM grids — all resulting from AWI absorption phenomenon of particles — have been well-recognized. However, the reasons for such misbehavior are not well-understood, limiting systematic approaches to addressing these problems. Here, we have proposed theoretical formulations that explain the AWI adsorption phenomenon and the associated problems. According to our formulation, the particles in the bulk solution migrate to the buffer surface to minimize the overall potential energy within the grid hole. As a result, the buffer surfaces become fully occupied by the particles. Based on this explanation, we experimentally examined the effect of surfactant application on the problems associated with the AWI adsorption phenomenon since surfactants can lower the surface energy $ {(\gamma }_{\mathrm{l}\mathrm{g}} $ ) by occupying the buffer surface to decrease the overall potential energy. To this end, we conducted cryoET to construct tomograms of the entire grid thickness to visualize the particle distribution in the hole of cryoEM grids. Indeed, our cryoET showed that all tested surfactants at CMC reduced the AWI adsorption of particles; FFC8 at CMC resulted in the most even distribution. Without surfactant, most particles were on the opposite surface to where the sample was applied. We conjectured that the particles began migrating to the opposite surface when the sample-applied surface was blotted, and that most particles had already finished migrating before the grid plunged, given that the particles can adsorb to the AWI in a matter of 0.1 s (Naydenova and Russo 2017; Noble *et al.* 2018a; Taylor and Glaeser 2008). Subsequent single-particle cryoEM confirmed that adding surfactant at CMC does not adversely affect the GroEL structure, as shown by near-atomic resolution reconstructions that are on par with those of the no-surfactant particles.

A question that arises naturally is what happens to the particles in the bulk volume, such as in the Eppendorf tube during long-term storage. Our theory suggests that particles would migrate to the surface until the surface is fully occupied by these particles. At this point, given the much smaller number of sites available on the surface area as compared to the bulk volume, the remaining particles would stay in the bulk volume, essentially eliminating the AWI problem for these particles. That is why the AWI adsorption problem has escaped observation until the advent of cryoEM, in which the particles are exposed to a greater surface and smaller bulk volume, a situation created by sample blotting. Our study rationalizes using surfactants in cryoEM sample preparation and experimentally demonstrates its effectiveness in lowering the surface energy $ {(\gamma }_{lg} $ ) and alleviating the AWI adsorption problem. However, potential concerns associated with the use of surfactants, such as micelle formation that could increase background noise and particle denaturation, still persist. Thus, other alternatives to surfactants are also worth exploring. For one, introducing a layer of 2D particle arrays on the AWI can be a barrier to protect other particles from AWI absorption (Glaeser and Han 2017). Another alternative is the new class of surfactants called amphipols (Liao *et al.* 2013; Tribet *et al.* 1996), which enable handling membrane proteins in a detergent-free solution. Recently, a support film using 2D crystals of hydrophobin HFBI was developed. The hydrophilic side of HFBI adsorbs sample particles via electrostatic interactions to protect them from the AWI, enabling thin ice formation for enhanced data collection (Fan *et al.* 2021). Other advanced engineering approaches aim to improve the cryoEM result quality without the use of surfactants. For example, a novel instrument has been developed to reduce the time before the sample plunges into liquid ethane (Noble *et al.* 2018b), and innovative support film (Han *et al.* 2020), like monolayer graphene-supported film and modified graphene/graphene oxides, have been developed to enhance cryoEM sample quality. More recently, *in situ* structures of ribosomes and expressomes were solved inside microbial cells via cryoET (Hoffmann *et al.* 2022; O'Reilly *et al.* 2020; Tegunov *et al.* 2021), avoiding the AWI problem altogether. However, at the time of this writing, such successes are still predominantly associated with large complexes such as ribosomes or ribosome-containing super-complexes. Consequently, high-resolution structures will continue to depend on practical solutions to resist or neutralize the potential energy at AWI, as discussed here through both theoretical consideration and experimental evidence.

## MATERIALS AND METHODS

### Sample preparation

Chaperonin 60 lyophilized powder (GroEL, 1 mg) from *Escherichia coli* was purchased from Sigma-Aldrich (Cat. #C7688, Sigma-Aldrich, St. Louis, MO, USA). GroEL powder was solubilized in 50 mmol/L Tris-HCl (pH7.5), 10 mmol/L KCl, and 10 mmol/L MgCl _2_. Subsequently, GroEL concentration was determined by NanoDrop Microvolume UV-Vis Spectrophotometers based on Protein A280. For short-term storage, 2 mg/mL GroEL was stored at 4 °C and used within two weeks. For long-term storage, stock solutions were stored at ‒80 °C and thawed before use.

### Grids preparation for cryoEM

GroEL solution was prepared at a concentration of 2 mg/mL and mixed with 1:70 diluted 5-nm diameter fiducial gold beads, in an 8:1 volume ratio. FEI Vitrobot Mark IV was used to make vitrified samples. The sample was applied to the carbon side of 200 mesh Cu Quantifoil 100 holey carbon films (R 2/1), which were beforehand glow discharged by Gatan Plasma System SOLARUS. Tested surfactants (concentrations shown in Table 1) were directly mixed with the sample right before freezing. The mixture was applied to the grid. The grids were blotted with filter paper to remove the extra sample and then plunged into liquid ethane. Grids were stored in liquid nitrogen.

**Table 1:** List of tested surfactants and their concentrations

Surfactant name	Surfactant concentration
No surfactant	Non-applicable
Fluorinated fos-choline 8	3.0, 0.3 and 0.03 mmol/L
DDM	0.17 mmol/L
NP40	0.3 mmol/L

### Single-particle cryoEM data collection

The grids were loaded into a Titan Krios electron microscope (Thermo Fisher Scientific) equipped with a Gatan imaging filter (GIF), and cryoEM images were recorded on a post-GIF Gatan K3 Summit direct electron detection camera operated in super-resolution electron-counting mode. The magnification was 81,000×, with a pixel size of 1.1038 Å/pixel at the specimen level. Data collection was facilitated by SerialEM (Mastronarde 2005). The dosage rate was set to 30 e ^−^/Å ^2^ at the sample level, and the exposure time for each frame was 0.2 s. The targeted under-focus value was 1.8–2.2 μm. In total, 500–700 micrographs were collected.

### Single-particle cryoEM data processing

We employed the computational steps outlined under the framework of WARP ( Tegunov and Cramer 2019) for the preprocessing, which included motion correction for the frame alignment and estimation of local defocus and resolution. Particle-picking was executed utilizing a machine learning algorithm (BoxNet) in WARP, generating, and yielding an average of 173,000 extracted particles with a box size of 400 pixels (pixel size: 1.1038 Å) for all conditions. The extracted particles were classified with the “2D Classification” tool on the Relion GUI with the following parameters: Number of classes, 32; Regularisation parameter T, 2; Number of iterations, 25; Mask diameter, 300 Å. After 2D classification, “good” class averages with clear top/side views of GroEL, as well as those that fit the general dimensions of GroEL were manually selected for 3D classification, using “Subset selection” tool on the Relion GUI. The 3D classification was conducted with the following parameters: Symmetry, D7; Number of classes, 4; Regularisation parameter T, 4; Number of iterations, 25; Mask diameter, 230 Å. After 3D classification, “good” class averages were manually selected for 3D refinement using the “Subset selection” tool on the Relion GUI. Particle images belonging to the selected “good” class averages were normalized to 20,936 particles. The normalized particles were subjected to a D7 symmetry refinement with a mask diameter of 200 Å, using “3D auto-refine” on the Relion GUI. Subsequent fitting of the crystal structure and images were created in Chimera X (Goddard *et al.* 2018).

### CryoET data collection

Single particle micrographs were collected on the FEI Tecnai TF20 at 200 Kilovolt (kV) equipped with a TIETZ F415MP 16-megapixel CCD camera. TEM Imaging & Analysis (TIA) was used to acquire data. All tilt series were collected from –50° to +50°, with 2° increments between each angle, using the FEI TEM Batch Tomography software. The nominal defocus was set to –6 μm. The cumulative dose count was 50–60 e ^−^/Å ^2^ per tilt series. The pixel size was 4.4 Å/pixel at the specimen level with the 50,000× nominal magnification for imaging.

### CryoET data processing

The tilt series were reconstructed by the Etomo component of the IMOD software package (Kremer *et al.* 1996) to 3D tomograms. “Build Tomogram” was chosen to start the reconstruction process. Tilt series images were pre-processed by removing the outlier pixel values in the data files. Coarse alignment was done using the fiducial seeding model, followed by a fine alignment. 10 to 15 gold beads were picked for each tilt series, and a mathematical model for specimen movements was used to predict the gold beads’ positions. The mean residual error was reduced during fine alignment by fixing big residuals. Positioning tomogram thickness was set to 1000 nm to include the top and bottom AWI. The tilt axis and Z shift were also computed and adjusted to create the final alignment. The final tomograms were built using SIRT with six iterations. Finally, the whole tomogram was rotated around the *x*-axis to make the air–water interface roughly parallel to the field of view.

GroEL particles were selected and saved as a mod file. Three points on the same plane were used to identify the AWI and the middle plane. The coordinates of all particles were recorded and used to calculate the distance to each plane. Particle distribution was visualized as a 3D model by IMOD.

## Conflict of interest

Joon S. Kang, Xueting Zhou, Yun-Tao Liu, Kaituo Wang and Z. Hong Zhou declare that they have no conflict of interest.
